# Development of a Fiber Laser with Independently Adjustable Properties for Optical Resolution Photoacoustic Microscopy

**DOI:** 10.1038/srep38674

**Published:** 2016-12-08

**Authors:** Esra Aytac-Kipergil, Aytac Demirkiran, Nasire Uluc, Seydi Yavas, Tunc Kayikcioglu, Sarper Salman, Sohret Gorkem Karamuk, Fatih Omer Ilday, Mehmet Burcin Unlu

**Affiliations:** 1 Department of Physics, Bogazici University, 34342, Istanbul, Turkey; 2 Institute of Materials Science and Nanotechnology, Bilkent University, 06800, Ankara, Turkey; 3 FiberLAST, Inc., 06800, Ankara, Turkey; 4 Department of Electrical and Electronics Engineering, Bilkent University, 06800, Ankara, Turkey; 5 Lumos Laser, Ltd., 06500, Ankara, Turkey; 6 Department of Physics, Bilkent University, Ultrafast Optics and Lasers Group, 06800, Ankara, Turkey

## Abstract

Photoacoustic imaging is based on the detection of generated acoustic waves through thermal expansion of tissue illuminated by short laser pulses. Fiber lasers as an excitation source for photoacoustic imaging have recently been preferred for their high repetition frequencies. Here, we report a unique fiber laser developed specifically for multiwavelength photoacoustic microscopy system. The laser is custom-made for maximum flexibility in adjustment of its parameters; pulse duration (5–10 ns), pulse energy (up to 10 μJ) and repetition frequency (up to 1 MHz) independently from each other and covers a broad spectral region from 450 to 1100 nm and also can emit wavelengths of 532, 355, and 266 nm. The laser system consists of a master oscillator power amplifier, seeding two stages; supercontinuum and harmonic generation units. The laser is outstanding since the oscillator, amplifier and supercontinuum generation parts are all-fiber integrated with custom-developed electronics and software. To demonstrate the feasibility of the system, the images of several elements of standardized resolution test chart are acquired at multiple wavelengths. The lateral resolution of optical resolution photoacoustic microscopy system is determined as 2.68 μm. The developed system may pave the way for spectroscopic photoacoustic microscopy applications via widely tunable fiber laser technologies.

Photoacoustic microscopy (PAM) is a promising imaging modality that combines optical and ultrasound imaging. It takes advantage of high optical contrast and high ultrasonic spatial resolution owing to its hybrid nature. When a short laser pulse illuminates tissue, absorbed light leads to acoustic emission via thermoelastic expansion^1^,^2^,^3^,^4^,^5^,^6^,^7^,^8^,^9^,^10^. Generated ultrasonic waves are conventionally detected by transducers. Recorded signals are used to map the distribution of the locations of optical absorbers. Relatively low scattering of ultrasonic waves in biological tissues provides deeper penetration beyond the optical transport mean free path^4^. The contrast of PAM is endogenously produced by optical absorption of chromophores within the tissue^11^,^12^.

The laser system needs to produce short enough pulses, *i.e.*, several nanoseconds, in order to generate photoacoustic signals efficiently and emit wavelengths in the visible range to cover absorption peaks of tissue chromophores in their spectra^4^,^13^,^14^. To obtain adequate penetration depth, it is also desirable to utilize a wavelength in the near infrared range, from 600 to 1200 nm, where biological tissues are relatively transparent^15^,^16^.

Several kinds of lasers have been used for photoacoustic imaging. Pulsed laser diodes draw researchers’ attention by being compact and inexpensive. While the peak power is relatively modest^15^,^17^, it is sufficient to obtain adequate signal-to-noise ratio for *in-vivo* optical resolution photoacoustic microscopy (OR-PAM), as demonstrated in several publications^18^,^19^,^20^,^21^,^22^. On the other hand, they found only limited place in photoacoustic applications due to their lack of continuous tunability in wavelength. Q-switched Nd:YAG lasers operating at 1064 nm (and/or acquiring 532 nm by frequency doubling) are frequently utilized for PAM^15^,^23^. They are generally preferred because of their easy accessibility. However, their fixed wavelength output is a serious drawback for multispectral photoacoustic applications which quantify unique spectral features of each absorber by a set of wavelengths. On the other hand, Q-switched Nd:YAG pumped dye lasers, Ti:Sapphire lasers, and optical parametric oscillators (OPOs) are usually preferred for providing necessary wavelength tuning with high pulse energies (>1 mJ)^7^,^8^,^14^,^24^,^25^,^26^,^27^,^28^,^29^,^30^,^31^,^32^; yet, they have some major limitations of their practical applications such as having low pulse repetition rate (generally less than 50 Hz, recently up to several kHz for OPOs^33^,^34^), being bulky and expensive, and requiring external cooling units^35^.

For the sake of enabling spectroscopic measurements, multiwavelength spectrum is obtained from a single wavelength emitting Q-switched Nd:YAG microchip laser, either through stimulated Raman scattering (SRS) or nonlinear broadening by coupling its output to a fiber^36^,^37^,^38^,^39^,^40^,^41^,^42^,^43^,^44^,^45^. For lasers utilizing SRS, major energy is distributed on a series of fixed individual wavelength peaks that result from nonlinear interaction between incoming photons through the fiber and the molecules in the fiber itself, thus offers a limited wavelength tunability^46^. Koeplinger *et al*.^41^ reported four bands in a polarization maintaining single mode fiber (PM-SMF), and Loya *et al*.^40^ improved the system with a broader wavelength tuning range also with a higher repetition rate and pulse energy per band. It was also demonstrated that both discrete lines and a continuum can be produced by using four-wave mixing in a special fiber (SMF-28e)^42^. As a different technique, Buma *et al*.^43^ used a birefringent optical fiber and produced discrete spectral bands in near infrared region. Much broader wavelength tuning can potentially be achieved by a supercontinuum source such as photonic crystal fiber (PCF), which relies on spectral broadening through nonlinear processes^36^,^47^,^48^,^49^. PCF is a silica optical fiber with an ordered array of microscopic air holes running along its length^50^,^51^. Billeh *et al*.^36^ utilized PCF for developing spectroscopic photoacoustic microscopy system. Lee *et al*.^37^ also built a supercontinuum laser system for both PAM and optical coherence tomography (OCT). Afterwards, Lee *et al*.^38^ determined oxygen saturation of hemoglobin and hemoglobin concentration via the same laser source. Whensoever the applications by coupling the output of Q-switched Nd:YAG microchip to PCF are considered, energy per band is reported to be lower in supercontinuum case than SRS, which may be a drawback for many applications^46^. In order to achieve wider tunability in the wavelength with high energy per band, Shu *et al*.^39^ proposed a master oscillator power amplifier (MOPA) laser system with a homebuilt yterrbium-doped (Yb) fiber amplifier for power boost. The amplifier was coupled to a specially designed PCF taper that connects a large-core fiber that has a much more resistance to high-pulse energy at the input to a small-core PCF for spectrum broadening. Pulse energy per band increased dramatically and became comparable to the ones produced through SRS^39^,^45^.

Apart from wavelength tunability, a laser system with high pulse repetition frequency (PRF) is also desired for fast image acquisition. The repetition frequencies of solid-state lasers are limited up to several kHz; but recently, fiber lasers with high repetition rates emerge as an alternative excitation source for PAM. Through their high repetition rate, near real and real time imaging can be achieved^46^,^52^,^53^,^54^. It has already been reported that in comparison to conventional systems with solid state lasers, the ones with fiber lasers are at least two orders of magnitude faster without compromising lateral resolution^52^,^55^. Fiber laser sources are also used for *in vivo* and *in vitro* studies also including flow cytometry applications^52^,^54^,^55^,^56^,^57^,^58^. The main disadvantage of these systems is their fixed wavelength that does not allow for multispectral functional imaging. To overcome the limitations, fiber laser technology seeking for tunability in wavelength is put forward. Hajireza *et al*.^59^ developed an SRS fiber laser source for photoacoustic imaging. They coupled the output of an Yb fiber laser into a PM-SMF in varying lengths at different PRFs and extended the number of wavelengths at SRS peaks that were previously limited^46^,^60^. In recent years, due to high power capabilities, MOPA laser systems have begun to be developed^61^,^62^,^63^. The first demonstration of a short pulse MOPA fiber laser at 1 μm was the study by Ilday *et al*.^64^. Allen *et al*.^61^ produced a fiber laser system with a high repetition frequency in MOPA configuration with a single emission wavelength of 1064 nm. Mahmud *et al*.^62^ demonstrated an OR-PAM system by using a commercial picosecond MOPA laser system consisting of a fiber-based tunable oscillator and three amplifier stages with a high power booster amplifier. However, the wavelength tunability was limited with 50 nm bandwidth.

Here, to address the limitations of each approach, we develop a tunable fiber based MOPA laser system producing nanosecond pulses, covering spectrum from 450 nm to 1100 nm, specifically for PAM. The supercontinuum part is all fiber-integrated; guided-beam-propagation renders its misalignment free and largely immune to mechanical perturbations. Free space harmonic generation creates higher pulse energy for a specific band, i.e. 532 nm, and also generates ultra violet (UV) light with wavelengths of 355 and 266 nm. Total supercontinuum output power is over 1 W and visible output power is around 270 mW at 65 kHz repetition rate corresponding to 4 μJ pulse energy. One of the novelties here is the improvement of wavelength tunability, output power and pulse energy when fiber-based lasers are benchmarked. This is the first demonstration of spectroscopic PAM by developing a supercontinuum all-fiber based MOPA source. The tunability of the laser parameters allows using only one laser for many different PAM applications, and also high repetition rate enables fast scanning. The coverage of near-UV spectrum gives an opportunity to image cell nuclei. As certain morphological changes such as size and shapes irregularities in the nuclei are known indicators of various cancers^30^,^65^,^66^, we believe our system may also be useful for cell nuclei studies as well.

## Results

**Figure 1:**
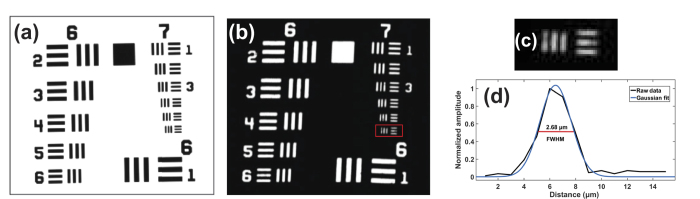
(**a**) Optical microscopy image, (**b**) Photoacoustic microscopy image of USAF resolution test target (Group 6 and 7). (**c**) Photoacoustic microscopy image of Group 7 Element 6. (**d**) FWHM of a line at Group 7 Element 6 from Gaussian fit (blue) of raw data (black).

A standardized resolution test target (USAF-1951, Thorlabs) was imaged for determination of the lateral resolution of our OR-PAM system. A transducer (V384, Panametrics) with a 3.5 MHz center frequency was used to acquire photoacoustic signals at the optical wavelength of 1064 nm filtered from the supercontinuum output. For focusing the light, a 5× objective (LMH-5 × −1064, Thorlabs) was used. The target was immersed in water, then 2D raster scanning by a motorized linear translation stage (LNR50SEK1, Thorlabs) along the *x-y* plane in steps of 1 μm for an area of 300 × 330 μm^2^ was performed. The acquired signals were averaged over 128 consecutive signal cycles. The trigger signal from the field programmable gate array (FPGA) of the laser was used to trigger a data acquisition card (DAQ) for synchronization. Following the triggering of each laser pulse, photoacoustic signals were initially amplified by 40 dB using a pre-amplifier (5678, 40 MHz bandwidth, Olympus) and then 39 dB via a pulser/receiver (5073PR, Olympus). The signals were digitized through a DAQ (Razor Express CompuScope 1422, Gage Applied Technologies, Inc.), then data processing and reconstruction were performed. Figure 1a shows the optical microscopy image and Fig. 1b presents the maximum amplitude projection (MAP) image of the scanned area (Group 6 and 7) of the test target. The lateral full width at half-maximum (FWHM) value from the imaged highlighted well resolved bars (Group 7, Element 6) was determined as 2.68 μm, as shown in Fig. 1d.

**Figure 2:**
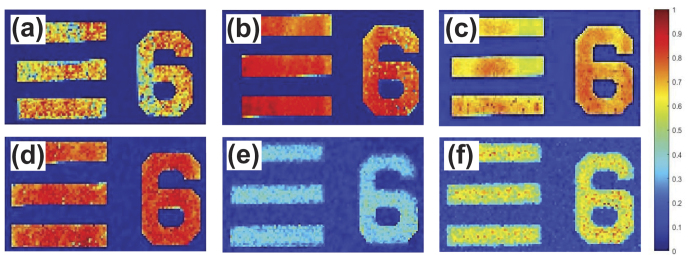
The PA image of Group 5 Element 6 scanned within an area of 56 × 101 μm^2^ with steps of 1 μm acquired at optical wavelength (**a**) 532 nm from harmonic generation unit, (**b**) 650 nm, (**c**) 697 nm, (**d**) 732 nm, (**e**) 785 nm, and (**f**) 880 nm, respectively from supercontinuum output.

Furthermore, for the demonstration of our multiwavelength PAM system, Group 5 Element 6 of the test target were also imaged with six different wavelengths of 532, 650, 697, 732, 785, and 880 that can be seen in Fig. 2a,b,c,d,e and f, respectively. These wavelength values except from 532 nm which was obtained by second harmonic generation (SHG), were filtered from the supercontinuum output of the laser for each experiment. A 10× objective (Plan Achromat, 0.25 NA, Olympus) was used to focus light to the relevant area.

## Discussion

In order to evaluate the performance of our laser system, pulse energy, average power and repetition rate values are compared with the ones in existing systems within the literature including fiber components and independent of seeding laser type. Billeh *et al*.^36^ sent the output of a Q-switched Nd:YAG microchip laser with a repetition frequency of 6.6 kHz to a 7 m-long PCF and reported seven wavelengths of 575, 625, 675, 725, 775, 825, and 875 nm with a bandwidth of 40 nm for each wavelength and pulse energies were measured as 7, 15, 24, 31, 31, 31, and 33 nJ, respectively. Lee *et al*.^38^ also sent the output of the same type of laser to a 10 m-long PCF and stated pulse energy of the generated supercontinuum light as 500 nJ. The pulse energies of two bands, 500 to 560 and 560 to 660 nm were measured as 0.6 and 1.8 nJ, respectively. As can be seen, these pulse energies are quite low despite the wide bandwidths. There are many attempts to develop multiwavelength laser systems generating higher pulse energies from the output of an integrated fiber for various photoacoustic imaging applications^36^,^39^,^40^,^41^,^45^. However, this condition requires PCFs to withstand such high energies. Since non-linearity increases as the effective mode area of fiber gets smaller; thus, it is advantageous to decrease core diameter for generation of more efficient supercontinuum. Yet, energy per surface area of the fiber has a major effect on the maximum optical pulse peak power which a fiber can withstand^67^,^68^,^69^. Therefore, there is a trade-off between supercontinuum efficiency and energy to be coupled into the fiber. In order to overcome this limitation, tapered fibers are designed. Bondu *et al*.^45^ used a nonlinear fiber that combines a large-core fiber for high-pulse energy handling with a small-core fiber for efficient spectral broadening. They used five different PCFs with varying core diameters, two of them were tapered for supercontinuum generation. They also demonstrated that total energy at the output of the straight PCF with core diameters of 5, 9, and 10 μm as 10, 29.5, and 30 μJ, respectively with visible output energies of 1.7, 5.4, and 4.6 μJ. Total output energy of tapered PCF of length of 1 m with an input core diameter of 10 μm tapered down to 5 μm was stated as 22 μJ with visible output energy of 6 μJ^39^,^45^.

Taking advantage of SRS inside a fiber is another method to increase the number of wavelengths from a fixed wavelength output. Polarization-maintaining single-mode fiber (PM-SMF) as well as PCF have been used for generation of SRS peaks^40^,^41^,^46^,^59^,^60^. Koeplinger *et al*.^41^ sent the output of a Q-switched Nd:YAG microchip laser with a repetition frequency of 7.5 kHz to a frequency-doubling KTP crystal. Then, this output was sent to a 6 m-long PM-SMF and acquired four distinct bands 546, 560, 574, and 600 nm with a pulse energy of 80 nJ for the each wavelength. Loya *et al*.^40^ coupled the output of a Q-switched Nd:YAG laser operating at 30 kHz repetition rate to a 30 m-long large mode area photonic crystal fiber (LMA-PCF) and individual pulse energies were reported as 270, 360, 520, 530, and 400 nJ at wavelengths of 532, 546, 568, 589, and 600 nm, respectively. Hajireza *et al*.^46^,^59^,^60^ coupled an Yb-doped fiber laser into a PM-SMF in varying lengths at different PRFs and extended the number of wavelengths at SRS peaks. The acquired pulse energies were in between 100 to 500 nJ.

Our tunable fiber-based laser system has three outputs; supercontinuum (from 450 to 1100 nm), 1064 nm from single-wavelength emitting port, and harmonic generation (532, 355, and 266 nm). The average power of 1064 nm output is around 3 W which seeds harmonic generation unit but also can be used for its own applications. The maximum average power values of SHG (532 nm), third harmonic generation (THG, 355 nm), and fourth harmonic generation (FHG, 266 nm) are 500, 3, 10 mW, respectively. Total output power of supercontinuum is measured over 1 W with visible output power around 270 mW with a powermeter (S314C, Thorlabs) at 65 kHz repetition rate that corresponds to 17 μJ total and 4 μJ visible energy. Various bandpass filters are used to obtain wavelength of interest from supercontinuum output and power measurements are performed to compare with the values in the literature. In order not to damage bandpass filters, a 1000 nm shortpass filter is firstly employed. Average power values at wavelengths of 680 and 830 nm with 10 nm bandwidths are measured as 5 and 11 mW by a powermeter (S142C, Thorlabs) after the achromatic lens that corresponds to 76 and 169 nJ pulse energy. For wider bandwidths, average power values for wavelengths of 650, 697, 732, 785, and 880 nm with 80, 75, 68, 62, and 70 nm bandwidths are 92, 93, 82, 84, 142 mW, respectively. Corresponding pulse energies are 1.4, 1.4, 1.3, 1.3, 2.2 μJ. These energies are higher than the ones produced through coupling the output of Q-switched Nd:YAG microchip laser to PCF which is at most 33 nJ^36^. As mentioned above, for the special case of tapered PCFs, visible output energy was reported as 6 μJ at 25 kHz, for our system that is 4 μJ at 65 kHz and comparable to that output^39^,^45^. In addition to that, our laser source can provide higher pulse repetition rate, up to 1 MHz, at the expense of lower pulse energies. For the systems utilizing SRS, the energies per band were reported several hundreds of nJ with an utmost energy of 500 nJ^46^,^60^. SRS peaks are produced with a bandwidth around 10 nm, pulse energies are higher than our system for such narrow bandwidths for visible region. However, when filters with wider bandwidths are selected, pulse energies become higher than ones that SRS peaks possess. To be also noted, pulse energies of SRS peaks decreases (estimated around 100 nJ) elongating near-infrared spectral region. The edge of peaks was noted as 788 nm^46^, our spectrum covers up to 1100 nm. Allen *et al*.^61^ produced an all-fiber laser source with a PRF up to 2 MHz but the output wavelength was fixed. Mahmud *et al*.^62^ also reported a fiber based laser source. By means of electronic modulations in the oscillator, tuning the repetition rate (0.1–120 MHz), the pulse-width (0.1–5 ns) and the wavelength (1030–1080 nm) were carried out. Green light was also generated through frequency doubling. The output power was reported up to 1.1 W and pulse energy up to 500 nJ. However, wavelength cannot be tuned in a broad range which does not allow for various spectroscopic photoacoustic applications. There are many other advantages of our system. All the laser parameters, which are reported as independently adjustable, could be achieved by changing FPGA configuration and currents to the pump diodes electronically without any mechanical intervention. The only exception to this is the switching among the supercontinuum and harmonic generation ports, which is achieved by a mechanically switchable mirror, that can also readily be motorized, if desired. In addition to this, it is very compact with dimensions of 40 × 40 × 9 *cm*^3^ except from free-space harmonic generation unit and does not require any big cooling unit. Thanks to its high PRF, it may be a promising source for cytometry as well^57^.

In our system, the light is transmitted through the splice between Yb-doped fiber and PCF for rendering all-fiber integrity with an efficiency of 40%. One of the disadvantages of current configuration is the heating at the splice point. Despite the cooling fan, the splice should be renewed once in a while in order to compensate for decreasing power in time. In order to handle the issue for robust and long-term operation, the splicing between the gain fiber and the PCF is optimized for low-loss and high tensile strength (using GPX-3000 series splicer, Vytran, Inc.), as demonstrated in the context of *in-situ* absorption spectroscopy of plasmas using a similar supercontinuum source and the same type of fibre^70^. Free space coupling is also possible between Yb-doped fiber and PCF; in that case transmission can be performed with higher efficiency and higher pulse energies can be produced if all-fiber integrity is disregarded. The present limitations to the continuously and independently adjustable laser parameters arise from the requirement of simultaneous satisfaction of the following conditions during laser design: ensuring that each amplification stage is seeded with sufficient power to prevent generation of laser noise in the form of amplified spontaneous emission (ASE), ensuring that the targeted, final pulse duration will depend on the seed pulse duration in a complex manner due to gain saturation and that there is sufficient peak power to accomplish the supercontinuum generation in the PCF. We believe that even a large range of parameters are possible, albeit at the cost of increased system complexity (by adding a second AOM and additional amplifier stages). The present parameter range was decided based on the balance between system complexity and sufficiency for most typical OR-PAM applications.

To sum up, when all-fiber based laser systems are taken into consideration, the developed system improves the wavelength tunability with a repetition rate up to 1 MHz. For laser systems having fiber components, pulse energies of this system are higher from PCF coupled supercontinuum cases and comparable to the outputs of special tapered PCF designs. The system also offers all-fiber integrity and higher PRF by means of custom developed FPGA electronics that controls laser diode. Pulse energies of SRS peaks can be surpassed at near-infrared region with same bandwidth, at visible region only by using filters with wider bandwidths. This paper presents the potential of a tunable fiber laser system in MOPA configuration for multiwavelength OR-PAM. We believe that the system may provide the means of spectroscopic photoacoustic microscopy applications via widely tunable fiber laser technologies.

## Methods

**Figure 3:**
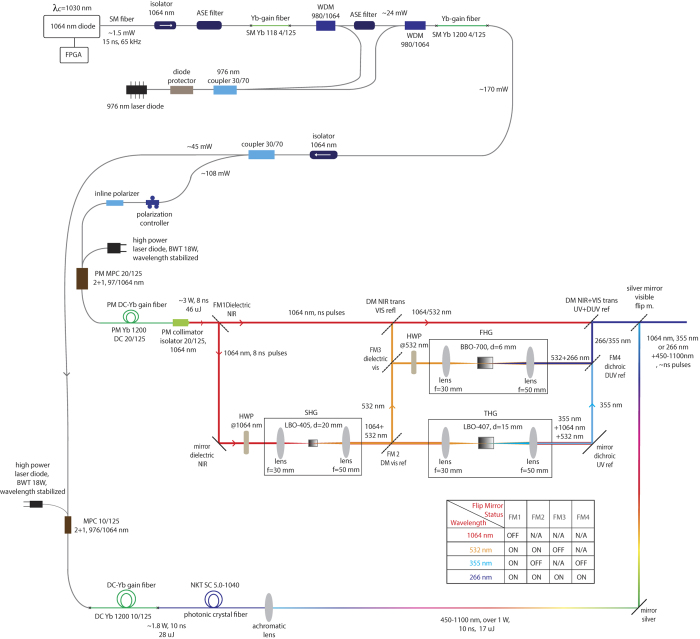
Schematics of fiber laser in MOPA configuration, all-fiber supercontinuum, and free-space harmonic generation units.

For photoacoustic microscopy system, a widely tunable fiber laser system is designed in MOPA configuration. Figure 3 shows the general scheme of the laser system. The output of MOPA configuration seeds two arms; the first one is used for supercontinuum generation via spectrum broadening and the second is for harmonic generation through nonlinear crystals. Pulses with sufficiently narrow bandwidths are required for harmonic generation (second, third, and fourth)^71^ through nonlinear crystals. The increase in the length of the crystal results in more efficient wavelength conversion; yet, longer crystals bring along phase shifts proportional to the bandwidth of the laser, and decrease the efficiency^72^. For this reason, a 1064 nm fiber-coupled diode laser (I-IV Laser Enterprise) with a very narrow bandwidth (0.3 nm) is used and driven by a nanosecond diode driver (PicoLas, LDP – V03–100 UF V3). Pulse width of the laser diode is adjusted through a field programmable gate array (FPGA) card (BASYS2, Xilinx). 15 ns long pulses at 65 kHz repetition rate are generated and sent to Yb-doped gain fiber after passing through an isolator and an amplified spontaneous emission (ASE) filter. As a pump source, a 976 nm laser diode (II-VI Laser Enterprise) delivering a maximum power of 540 mW is used. The pump is first passed through a pump protection filter with a maximum power handling of 300 mW, followed by a 30:70 coupler allotting two stages of preamplifier. In the first stage, Yb-doped fiber is backward-pumped by 30% of the output of the laser diode, then combined with the signal through a wavelength division multiplexer (WDM). Backward pumping is crucial for decreasing ASE generation rate, and hence preventing possible damage to the pump diodes and other fiber components. Another ASE filter is used between pre-amplifier stages to prevent the first from ASE that may be produced in the second. A WDM is used to combine 70% of the output of the laser diode pump and the first stage of the preamplifier. For amplification, an Yb-doped fiber is used and the output power is measured as 170 mW at 65 kHz repetition rate. The last component of the second preamplifier is an isolator with a maximum power handling of 2 W in order to protect it from back reflections.

**Figure 4:**
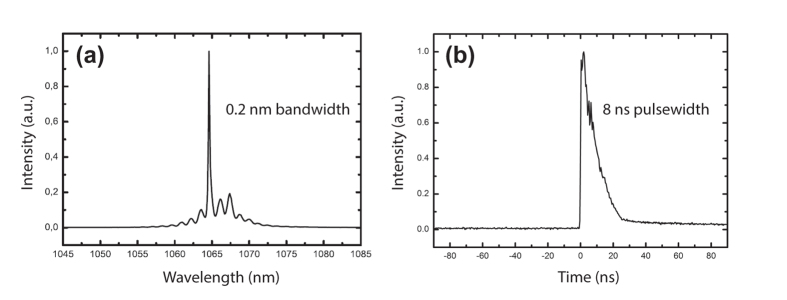
(**a**) Optical spectrum, (**b**) Temporal profile of a typical pulse at the end of PM-DC-Yb fiber.

**Figure 5:**
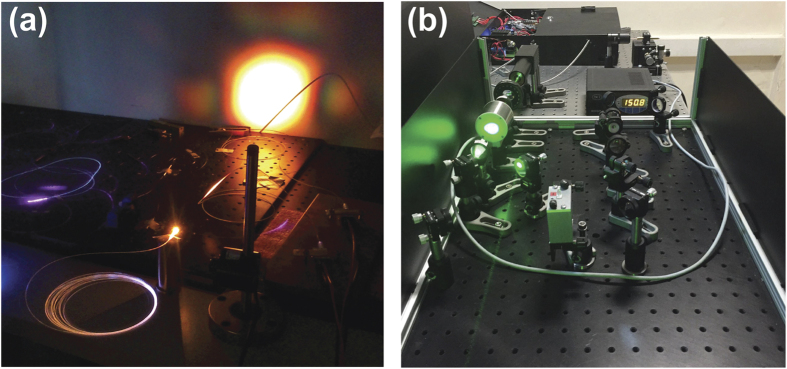
Photographs of the outputs of (**a**) supercontinuum, and (**b**) harmonic generation unit.

**Figure 6:**
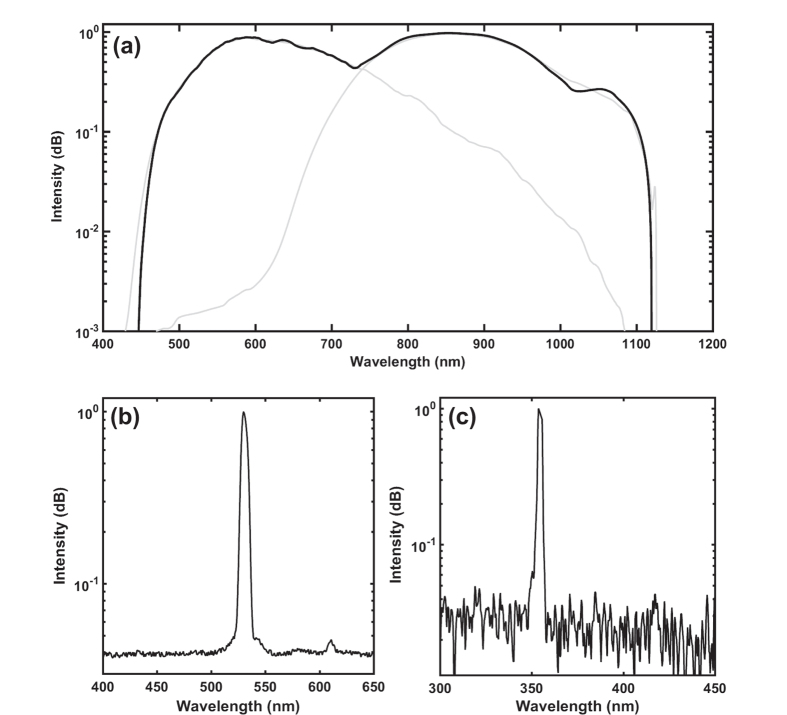
Optical spectrum of the (**a**) supercontinuum output (acquired by OSA 1 and OSA 2, respectively), (**b**) SHG (acquired by OSA 2), and (**c**) THG (acquired by OSA 1).

At the end of the preamplifier, a 30:70 coupler separates the signal, 30% is utilized for supercontinuum and 70% is for harmonic generation. Polarization of light is crucial for frequency multiplication; thus, 70% of the allocated signal is passed through a polarizer and all fiber components beyond this point are polarization maintaining. A 976 nm diode laser is used and a multi-mode pump combiner (MPC) combines the pump and signal. A polarization maintaining double cladding Yb-doped (PM-DC-Yb) fiber is spliced to the end of the MPC for amplification of the signal and pulses with 8 ns duration with an average power of 3 W at 65 kHz repetition rate are acquired. Figure 4a shows the optical spectrum and Fig. 4b shows the temporal profile of a pulse at the end of the amplification. In the temporal profile, the leading edge of the pulse is sharpened, or self-steepened, as the gain is partially saturated by each individual pulse and consequently less gain is available for the trailing edge. The temporal structure in the trailing edge is a static structure, which does not vary from pulse to pulse, originating primarily from the dynamically varying impedance of the semiconductor diode that seeds the system. Besides, 30% of the signal having an average power of 45 mW is firstly amplified for supercontinuum generation, a 15 m long PCF (SC 5.0–1040, NKT) with 5 μm core size is spliced to the end of Yb-doped fiber (Yb-1200 20/125 PM, nLight Liekki). The core size of the Yb-doped fiber is 20 μm which is larger than the core size of the PCF. For this reason, a special splice is used in between the Yb-doped fiber and PCF by a suitable splicer (FSM-100M, Fujikura). Figure 5a and b show the photograph of the output of supercontinuum and harmonic generation units, respectively. Optical spectrum of the supercontinuum is measured by two optical spectrum analyzers (OSA) with different wavelength ranges; OSA 1 (Avaspec-3648-VIS, Avantes) and OSA 2 (QE65 Pro, Ocean Optics). The acquired spectra are digitally combined in a single figure (Fig. 6a). In the first spectrum, the intensity of near infrared region appears lower than its actual level due to the decrease in the response of the analyzer while approaching to the edges of the measurable spectra region. It may also be caused by the difficulty of collecting all the beam with broad spectrum which is collimated by a single lens. Although the lens is an achromatic lens, it may still not be enough to eliminate slight divergence for different wavelengths and thus amplitude measurement variation throughout this broad spectrum range. In the second one, the intensity of the region between 450 to 650 nm lowered to noise level as a result of using neutral density filters in order to prevent saturation of the detector for the remaining spectrum.

For frequency multiplication process, a half wave plate is employed to match the polarization between the isolator and crystals. An anti-reflection coated (for 1064 nm wavelength) lens with a focal length of 30 mm is used to focus light into crystal. For SHG, a 20 mm long Lithium Triborate (LBO) crystal (Eksma, LBO-405) is used. For non-critical phase matching (NCPM), a crystal oven and a proportional–integral (PI) controller is added to maintain the temperature at 150.8 °C that results in maximum power. The light is passed through an anti-reflection coated (for 532/1064 nm) lens for collimation. Two dichroic mirrors separate the generated SHG beam (532 nm light) from the 1064 nm beam. Here, the output power is measured as 500 mW for 532 nm light. A mirror hold including a dichroic mirror reflecting 532 nm wavelength is added to the system. When the mirror is flopped, beam including 532 and 1064 nm wavelengths pass through a lens to enter a crystal (Eksma LBO-407) for THG. The crystal is maintained at 40 °C for NCPM. The output power is around 3 mW for 355 nm light. Another flip mirror mount with a dichroic mirror that is transmitting 1064 nm and reflecting 532 nm beam is added to direct the beam toward a lens with a focal distance of 30 mm. This lens focuses the beam into a Barium Borate (BBO) crystal (Eksma BBO-700, thickness = 6 mm) that generates second harmonic of the 532 nm beam (fourth harmonic generation), which results in around 10 mW of 266 nm light. The output of the crystal is filtered via a dichroic mirror reflecting 266 nm light and collimated by using a UV-coated lens with a focal distance of 50 mm. The optical spectrum of SHG is shown in Fig. 6b and of THG in Fig. 6c. The spectra are acquired with OSA 2 and OSA 1, respectively. Figure 3 shows the schematics of fiber laser in MOPA configuration, all-fiber supercontinuum, and free-space harmonic generation units.

**Figure 7:**
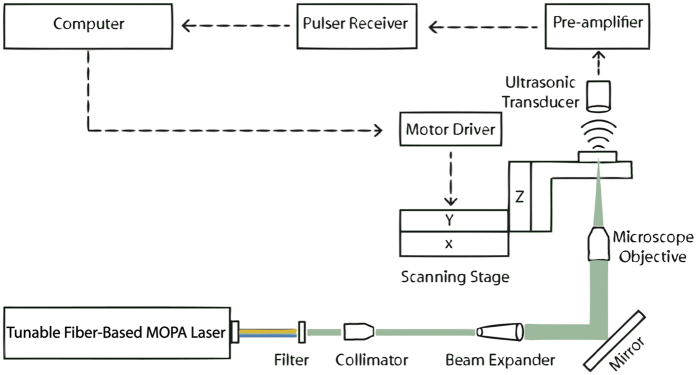
The schematics of experimental setup for transmission mode OR-PAM system.

The schematics of experimental setup for transmission mode OR-PAM system by using the irradiation source explained previously is shown in Fig. 7. Pulse duration of the laser is 8-ns for harmonics generation output and 10 ns for supercontinuum port.

## Additional Information

**How to cite this article**: Aytac-Kipergil, E. *et al*. Development of a Fiber Laser with Independently Adjustable Properties for Optical Resolution Photoacoustic Microscopy. *Sci. Rep.* **6**, 38674; doi: 10.1038/srep38674 (2016).

**Publisher's note:** Springer Nature remains neutral with regard to jurisdictional claims in published maps and institutional affiliations.
